# Exciton-photon correlations in bosonic condensates of exciton-polaritons

**DOI:** 10.1038/srep12020

**Published:** 2015-07-08

**Authors:** Alexey V. Kavokin, Alexandra S. Sheremet, Ivan A. Shelykh, Pavlos G. Lagoudakis, Yuri G. Rubo

**Affiliations:** 1 School of Physics and Astronomy, University of Southampton, SO 171 BJ Southampton, United Kingdom; 2 Russian Quantum Center, Novaya 100, 143025 Skolkovo, Moscow Region, Russia; 3 Spin Optics Laboratory, St.-Petersburg State University, 198504 Peterhof, St.-Petersburg, Russia; 4 Department of Theoretical Physics, St-Petersburg State Polytechnic University, 195251 St.-Petersburg, Russia; 5 Science Institute, University of Iceland, Dunhagi-3, IS-107, Reykjavik, Iceland; 6 Division of Physics and Applied Physics, Nanyang Technological University, 637371 Singapore; 7 ITMO University, 197101 St.-Petersburg, Russia; 8 Instituto de Energías Renovables, Universidad Nacional Autónoma de México, Temixco, Morelos, 62580 Mexico

## Abstract

Exciton-polaritons are mixed light-matter quasiparticles. We have developed a statistical model describing stochastic exciton-photon transitions within a condensate of exciton polaritons. We show that the exciton-photon correlator depends on the rate of incoherent exciton-photon transformations in the condensate. We discuss implications of this effect for the quantum statistics of photons emitted by polariton lasers.

A pulse of light entering a crystal in the vicinity of the exciton resonance excites combined light-matter waves, as it was discovered by Pekar in 1957^1^. Quantization of these waves yields mixed light-matter quasiparticles, currently known as exciton-polaritons. A classical theory developed by Hopfield in 1960s describes this process characterizing crystals by modified dielectric functions having resonant features^2^. The quantum theory developed by Agranovich^3^ considers a polariton as a chain of virtual emission-absorption acts: a photon is absorbed creating an exciton, then an exciton recombines emitting a photon, etc. It is usually assumed that neither emission nor absorption of photons by excitons does take place in reality: photons entering a crystal are immediately transformed into exciton-photon quasiparticles which may be detected as excitons or as photons with certain probabilities given by so-called Hopfield coefficients. It is impossible to know a priori if an exciton-polariton would collapse to exciton or a photon state in each particular measurement. Meanwhile, excitons in quantum confined potentials may be characterized by radiative lifetimes describing their spontaneous recombination with emission of photons^4^. This picture is referred to as a weak coupling regime, where the emission and absorption acts take place in reality, and notion of exciton-polaritons is of no much use.

The crossover between weak-coupling and strong-coupling regimes is illustrated in Fig. 1. It is manifested by the appearance of two distinct exciton-polariton resonances in the reflectivity or transmission spectra which are split in energy. The transition from weak- to strong-coupling is routinely described assuming the coherent exciton-light coupling combined with phenomenologically introduced broadenings of exciton and photon modes^5^. However, this approach fails to account for stochastic absorption and emission of photons by excitons, that are clearly dominant in the weak-coupling regime, e.g. for a single exciton resonance coupled to a continuum of photon modes^6^. There is no reason to expect that stochastic exciton-photon conversions disappear abruptly at the weak-strong coupling crossover.

The interpretation of exciton-polaritons as superposition light-matter quantum states is essential for understanding of the phenomenon of polariton lasing^7^. In polariton lasers, stimulated scattering of exciton-polaritons in semiconductor microcavities^8^ leads to macroscopic population of a single quantum state, which forms a condensate (or Bose-Einstein condensate^9^,^10^) of exciton-polaritons. A polariton condensate spontaneously emits the monochromatic and coherent light, which constitutes the polariton lasing effect^7^,^11^. Polariton lasers have been experimentally realized in various semiconductor systems^12^,^13^,^14^,^15^ and operate up to room temperature^16^,^17^. From the point of view of the quantum theory^18^, spontaneous emission of each individual photon does not collapse the many-body wave-function of the polariton condensate, which opens room for studying the internal structure of this mixed-light matter many-body state by measuring the statistics of emitted photons^19^,^20^,^21^. It is yet unclear if stochastic emission and absorption of photons by excitons plays any role in the strong coupling regime, but it seems highly likely that stochastic processes are important in the vicinity of weak-strong coupling transition and at the onset of polariton lasing. The coexistence of weak and strong coupling in semiconductor microcavities has been experimentally observed by Lagoudakis *et al.*^22^.

Here we present the model of exciton-polariton system describing both coherent and stochastic transformations of excitons and photons. Coherent processes are characterized by the Rabi frequency Ω, i.e., the splitting of upper and lower exciton-polariton eigenmodes, while the acts of stochastic conversion happen with a characteristic time *τ*_*xc*_. The exciton-photon conversion rate constitutes an important supplementary characteristic of the system. In order to reveal the role of this rate in the noise spectra of photoluminescence and photocurrent generated by exciton-polaritons, one can place a condensate of exciton-polaritons in a biased semiconductor microcavity structure^23^, where polaritons can decay as excitons by electron and hole tunneling through the barriers or as photons by photon tunneling through dielectric mirrors of the microcavity thus contributing to the photoluminescence signal. We quantitatively analyze the correlations between the noises in exciton and photon decay channels as functions of the stochastic exciton-photon and photon-exciton conversion rate 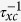. We discuss the potential impact of the stochastic processes on the second order coherence in the emission of polariton lasers and compare the results of our statistical model with the experimental data by Kasprzak *et al.*^24^.

## Basic equations

The strong exciton-photon coupling regime in microcavities manifests itself in the appearance of a Rabi doublet composed by lower (LP) and upper polariton (UP) branches. The lower 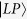 and upper 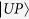 single-polariton states can be represented as


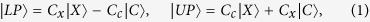


where 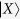 and 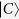 denote the exciton and cavity quantum states, respectively. The Hopfield coefficients are given by 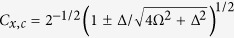 with Δ being the detuning between the bare photon and exciton mode^8^. In the following we shall restrict ourselves to the simplest Δ = 0 case.

The system of exciton-polaritons is described by the density matrix 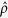, which evolves according to the equation


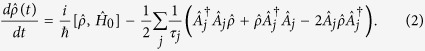


Here we assume that exciton-polaritons are excited at *t* = 0 and only decay processes are present at *t* > 0. The decay processes under consideration are described by four Lindblad terms with *j* = *x*, *c*, *xc*, *cx*. Denoting by 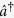 (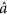) and 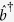 (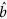) the creation (annihilation) operators for excitons and photons, respectively, we describe the escape (annihilation) of an exciton with the operator 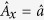 and the characteristic life-time *τ*_*x*_, the escape of a photon is described with 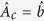 and the life-time *τ*_*c*_. The two processes of conversion are present: the exciton-to-photon conversion with 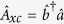 and the photon-to-exciton conversion with 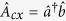, both having the same characteristic conversion time *τ*_*xc*_. We note that the stochastic conversion appears, in particular, if one considers the scattering of exciton and photons with some reservoir modes, in particular, with acoustical phonons. The conversion terms can be derived from the interaction Hamiltonian like 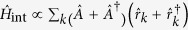 after averaging over the reservoir operators 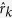 and 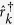 within the Born-Markov approximation. Finally, the equation for the density matrix is completed by the term describing the coherent exciton-photon coupling with


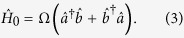


Before we proceed further, let us emphasize the limitations of the chosen model. First, it neglects the possible supplementary gain due to the stimulated relaxation of exciton-polaritons from an incoherent reservoir. The role of reservoir has been carefully discussed in the literature, e.g., by Wouters and Carusotto^25^. It is crucial for polariton lasers operating in the cw regime under non-resonant optical pumping. In this work we would like to consider a polariton condensate resonantly excited by a laser pulse. The reservoir in this case would not form. Second, we shall assume that the internal exciton-photon correlations in our condensate directly affect the output signal of the device under study (the photoluminescence, photocurrent or Kerr angle signals, in particular). We realise that this is not always the case, as has been pointed out by Cuiti and Carusotto^26^. We discuss the methods of experimental observation of exciton-photon correlations below.

Stochastic exciton-photon conversion makes evolution of 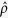 to be quite complex, as it can be seen from the Glauber-Sudarshan representation of Eq. (2). Expanding of 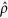 on the basis of coherent states 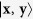, where **x** and **y** are 2D vectors such that 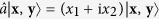 and 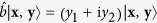, one can obtain


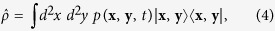



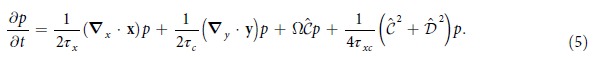


Here operator


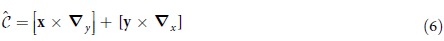


describes the Rabi oscillation in the system, while


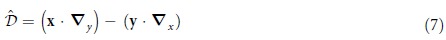


governs the stochastic mixing between exciton and photon subsystems.

The above equations describe the exciton-polariton system, which was excited at *t* = 0 and evolves freely afterwards. This is the most convenient set-up to study the effects of stochastic conversion between excitons and photons. We note that in the presence of cw pumping that results in formation of an incoherent exciton reservoir^25^, the density matrix equation (2) is modified by adding the gain term with 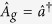 and 1/*τ*_*g*_ = *W*, where *W* is the exciton harvest rate from the reservoir to the exciton-photon system. This would lead to the term 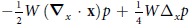 in the rhs of the Fokker-Planck equation (5).

The effect of conversion can be analyzed in the limiting case *τ*_*x*_, *τ*_*c*_ ≫ *τ*_*xc*_, where the first two terms in the right-hand-side of (5) can be omitted. The remaining Fokker-Planck equation conserves the average number of particles 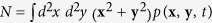, having in mind that 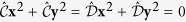. Moreover, any function *p*(**x**^2^ + **y**^2^) represents a time-independent solution. The system evolves to reach the state that corresponds to the equidistribution of exciton and photons, what also implies the equidistribution between upper and lower polariton branches.

The operators 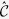 and 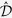 obey the following useful relations: 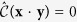, 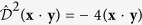. These relations can be used to obtain the time-dependence of the total energy of the system 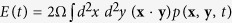, which decays exponentially to zero, which corresponds to the bare exciton (photon) energy in our notation, *E*(*t*) ∝ exp(– *t*/*τ*_*E*_), with


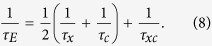


We note that for finite *τ*_*x*,*c*_ the average number of particles also decays exponentially, *N*(*t*) ∝ exp(– *t*/*τ*_*N*_), but on a different time scale


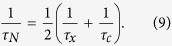


The stochastic conversion only transforms excitons to photons and vice versa and, therefore, does not affect the evolution of the total number of particles in the system. On the other hand, the conversion process does not conserve the energy and modify the energy evolution. As a result, the presence of stochastic conversion changes the energy per particle *E*/*N*, which is otherwise time-independent for *τ*_*xc*_ = 0

In what follows, several correlators of interest will be defined by the diagonal elements of the density matrix *P*(*n*_*a*_, *n*_*b*_, *t*) in the basis of exciton and photon number states, which describe the probabilities to have *n*_*a*_ excitons and *n*_*b*_ photons in the system. The dynamics of *P*(*n*_*a*_, *n*_*b*_, *t*) is found from (2) to be


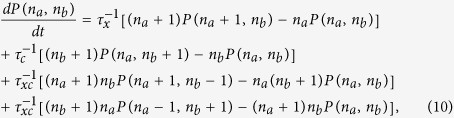


where the sum of the diagonal elements is normalized to unity 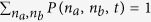.

## Results and Discussion

The quantities of interest to be analyzed in the remaining part of this Report include the exciton-photon correlator


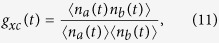


the exciton and photon second-order coherence


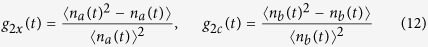


and the generalized exciton-photon correlator expressed through the previous three correlators


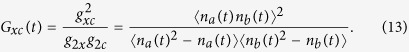


The latter correlator is convenient since *G*_*xc*_ = 1 for a polariton condensate formed at the low-polariton branch independently of the statistics of particles within the condensate. To prove this statement, we express the exciton and photon operators through the polaritons operators corresponding to the lower and the upper branches, 
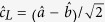
 and 
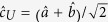
, respectively. Assuming that the upper polariton branch is empty, we obtain


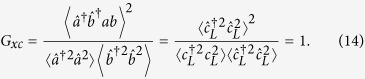


As we discussed above, the stochastic exciton-photon conversion leads to the mixing of lower and upper polariton branches, and this is indicated by deviation of *G*_*xc*_ from unity. We consider coherent and Fock states of polaritons at the lower polariton branch as initial states of our system. The initial diagonal elements of the density matrix for the coherent state can be described by the product of two Poisson distributions


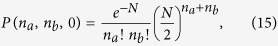


while for the Fock state they are given by


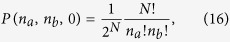


where *N* is the average number of the lower-branch polaritons at *t* = 0.

**Figure 2:**
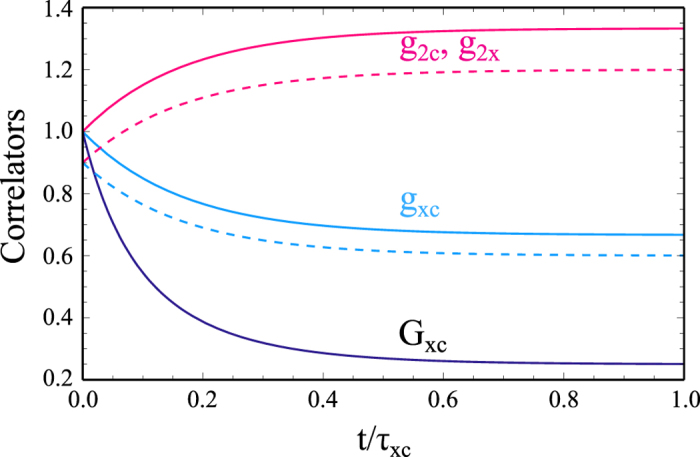
(Color online) The time evolution of the correlators *G*_*xc*_(*t*) (black), *g*_*xc*_(*t*) (blue) and *g*_2*x*_(*t*) = *g*_2*c*_(*t*) (red). There is initially *N*(0) = 10 lower-branch polaritons in the coherent (solid lines) and and the Fock (dashed lines) states (see text). Infinite lifetimes of excitons and photons *τ*_*x*_ = *τ*_*c*_ = ∞ are assumed.

Figure 2 shows the time evolution of the correlators *G*_*xc*_(*t*), *g*_*xc*_(*t*), *g*_2*x*_(*t*), and *g*_2*c*_(*t*), calculated assuming infinite exciton and photon lifetimes. As expected for the initial coherent state, *g*_*xc*_(0) = *g*_2*x*_(0) = *g*_2*c*_(0) = *G*_*xc*_(0) = 1. The subsequent change of all considered correlators is substantial and experimentally verifiable. On the time scale of *τ*_*xc*_ the correlators approach their saturation values,


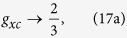



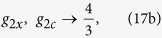



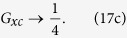


These saturations values (17a–c) are quasiclassical and can be obtained using the equidistribution of particles, i.e., using the density matrix (4) with the kernel *p* depending on **x**^2^ + **y**^2^ only. There are quantum corrections to the expressions (17a–c) where the kernel *p*(**x**, **y**) is a non-positive and singular function. Consider, e.g., the evolution of the initial Fock state of *N* polaritons in the simplest case of infinite exciton and photon lifetimes. The stochastic mixing process equalizes the probabilities of all possible combinations of *n*_*a*_ and *n*_*b*_ such as *n*_*a*_ + *n*_*b*_ = *N*. Namely,


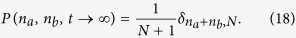


As a result, for *t* → ∞ we have 〈*n*_*a*,*b*_〉 = *N*/2, 
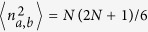
, 〈*n*_*a*_*n*_*b*_〉 = *N*(*N* – 1)/6, so that the factor (1 – *N*^−1^) appears in the right-hand-sides of the expressions (17a,b).

**Figure 3:**
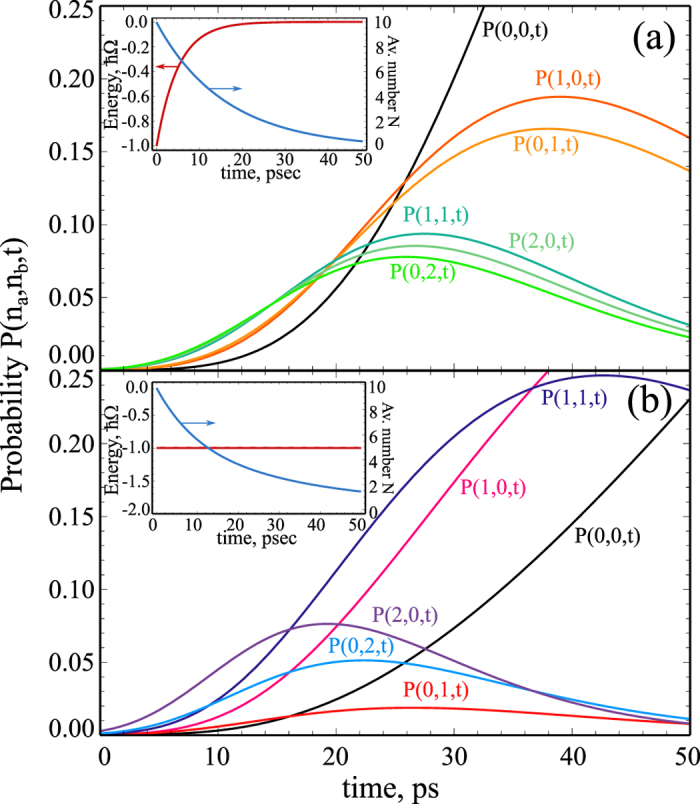
(Color online) Showing evolution of some diagonal elements *P*(*n*_*a*_, *n*_*b*_, *t*), as well as evolution of the average number of particles *N* and the energy per particle on insets. The panel (**a**) corresponds to the finite time of the exciton-photon conversion *τ*_*xc*_ = 5 ps, while the behavior in the absence of stochastic conversion is shown in the panel (**b**) The polariton condensate is initially in the coherent state with *N* = 10. The life-times of excitons and photons are *τ*_*x*_ = 40 ps and *τ*_*c*_ = 10 ps.

In Fig. 3 we demonstrate the behaviour of several diagonal elements *P*(*n*_*a*_, *n*_*b*_, *t*) of the density matrix as functions of time for the initial coherent distribution (15) and realistic exciton and photon lifetimes. In the panel (a) we account for the finite stochastic exciton-photon conversion time, while in the panel (b) the stochastic conversion is neglected. In the absence of stochastic processes there is an asymmetry in the behaviour of the density matrix elements *P*(*n*_*a*_, *n*_*b*_, *t*) corresponding to the same number of polaritons *N* = *n*_*a*_ + *n*_*b*_, which is caused by the difference of exciton and photon lifetimes. The stochastic process affects noticeably this behavior, as it is seen in Fig. 3(a). Due to the transformations of photons to excitons and vice versa, the density matrix elements *P*(*n*_*a*_, *n*_*b*_, *t*) ≈ *P*(*n*_*b*_, *n*_*a*_, *t*) if *t* > *τ*_*xc*_. The insets in Figs 3(a,b) show the calculated energy per particle. As expected, this energy exponentially tends to zero on the timescale of *τ*_*xc*_ [inset in Fig. 3(a)], while it is constant in the absence of the stochastic mixing [inset in Fig. 3(b)).

This analysis evidences a set of fingerprints of the stochastic processes which distinguish them from the conventional exciton or photon decay: the energy of the condensate blue-shifts with time, its broadening increases, the correlators *g*_*xc*_ and *G*_*xc*_ go below 1.

It is seen that the pronounced variations of the quantum coherence, exciton-photon correlators and the energy-per-particle with time may be observed if *τ*_*xc*_ < *τ*_*x*_, *τ*_*c*_. How short can be the exciton-photon conversion time *τ*_*xc*_ in realistic structures? Clearly, in the strong coupling regime one requires *τ*_*xc*_ > Ω^−1^, otherwise the polariton Rabi-oscillations^27^,^28^,^29^,^30^,^31^ would not be observed in time-resolved optical spectroscopy experiments. Most likely, *τ*_*xc*_ should be of the order of the decoherence time of Rabi oscillations (typically, 3–10 ps), which can still be short compared to the polariton lifetime. The most direct way to prove the existence of stochastic exciton-photon conversions in polariton condensates would be through the measurement of the generalized exciton-photon correlator (13). Deviation of this correlator from 1 would be a signature of the stochastic process described here. However, the experimental detection of correlations between photocurrent and photoluminescence is a challenging task. A simpler experiment would consist in simultaneous measurements of the noise in Kerr rotation angle of a linearly polarized probe pulse and the noise in the photoluminescence of a polariton condensate excited by a circularly polarized pump pulse. Indeed, the Kerr rotation angle is proportional to the exciton fraction of the polariton condensate^30^, while the photoluminescence signal is proportional to its photonic fraction. In this purely optical experiment one should make sure that the intracavity exciton-photon correlations of the condensate are directly traduced to the correlations between the linearly polarised Kerr and circularly polarised photoluminescence signal which may not be the case e.g. in the ultra-strong coupling regime^26^.

Note, that the stochastic exciton-photon conversion might be responsible for the increase of the second order coherence function *g*_2_(0) above the polariton lasing threshold in the experiments of Kasprzak *et al.*^24^. The experimental *g*_2_(0) increases even beyond the predicted by our model limit of 4/3. This may be a consequence of other mechanisms of decoherence, e.g. polariton-polariton scattering and interaction with bi-excitons.

## Conclusions

In this Report we have specifically considered an initial coherent state of the polariton condensate excited by a laser pulse, which is the most likely experimental scenario, and only confronted it to the exotic Fock state limit. Our theory can be easily extended to any other statistics of the initial state (e.g., the thermal statistics). Our goal is to show that the stochastic conversion of excitons into photons and vice versa should have strong influence upon the quantum optical properties of exciton-polariton condensates. The crucial parameter of the model is the exciton-photon conversion time *τ*_*xc*_. We expect it to be of the same order of magnitude as exciton and photon lifetimes *τ*_*x*_, *τ*_*c*_ at the onset of the weak couplig regime. *τ*_*xc*_ → ∞ limit is likely to be realised in the ultra-strong coupling regime. We expect that new experiments will shed light on the behaviour of exciton-photon correlators introduced in this work and improve our understanding of a unique light-matter superposition state: condensate of exciton-polaritons.

## Additional Information

**How to cite this article**: Kavokin, A. V. *et al.* Exciton-photon correlations in bosonic condensates of exciton-polaritons. *Sci. Rep.* **5**, 12020; doi: 10.1038/srep12020 (2015).

## Figures and Tables

**Figure 1:**
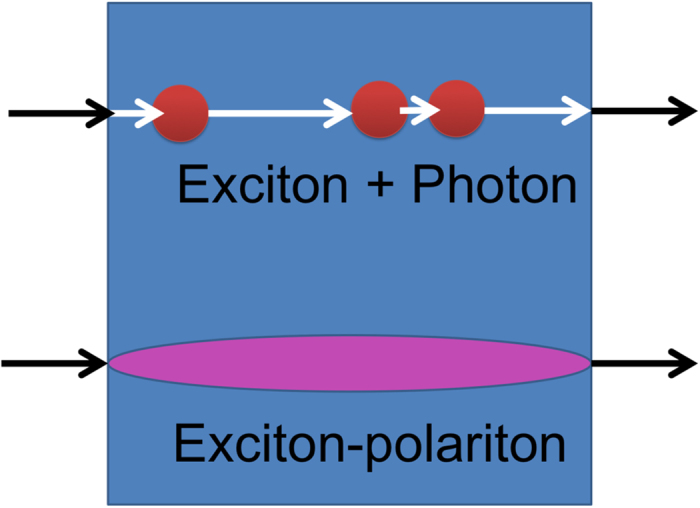
(Color online) The schematic of photon-exciton interaction in the weak coupling (upper panel) and strong coupling (lower panel) regimes. While in the weak coupling regime real absorption and emission of photons by excitons takes place, the strong coupling is characterised by appearance of a superposition exciton-photon state.

## References

[b1] Pekar S. I. Theory of electromagnetic waves in a crystal in which excitons arise. Zh. Eksp. Teor. Fiz. 33, 1022 (1957) [Sov. Phys. JETP **6**, 785 (1958)].

[b2] Hopfield J. J. Theory of the contribution of excitons to the complex dielectric constant of crystals. Phys. Rev., 112, 1555 (1958).

[b3] Agranovich V. M. On the influence of reabsorption on the decay of fluorescence in molecular crystals. Optika i Spectr. 3, 84 (1957).

[b4] Andreani L. C., Tassone F. & Bassani F. Radiative lifetime of free excitons in quantum wells. Solid State Communications. 77, 641 (1991).

[b5] Savona V., Andreani L. C., Schwendimann P. & Quattropani A. Quantum well excitons in microcavities: Unified treatment of strong and weak coupling regimes. Solid State Communications. 93, 733 (1995).

[b6] Lodahl P. *et al.* Controlling the dynamics of spontaneous emission from quantum dots by photonic crystals. Nature. 430, 654 (2004). 15295594 10.1038/nature02772

[b7] Imamoglu A., Ram R. J., Paul S. & Yamamoto Y. Nonequilibrium condensates and lasers without inversion: Exciton-polariton lasers. Phys. Rev. A. 53, 4250 (1996). 9913395 10.1103/physreva.53.4250

[b8] Kavokin A., Baumberg J. J., Malpuech G. & Laussy F. P. Microcavities (Oxford University Press, 2007).

[b9] Deng H., Haug H. & Yamamoto Y. Exciton-polariton Bose-Einstein condensation. Rev. Mod. Phys. 82, 1489 (2010).

[b10] Butov L. V. & Kavokin A. V. The behavior of exciton-polaritons. Nature Photonics. 6, 2 (2012).

[b11] Porras D., Ciuti C., Baumberg J. J. & Tejedor C. Polariton dynamics and Bose-Einstein condensation in semiconductor microcavities. Phys. Rev. B 66, 085304 (2002).

[b12] Kasprzak J. *et al.* Bose-Einstein condensation of exciton polaritons. Nature 443, 409 (2006). 17006506 10.1038/nature05131

[b13] Balili R., Hartwell V., Snoke D., Pfeiffer L. & West K. Bose-Einstein Condensation of Microcavity Polaritons in a Trap. Science 316, 1007 (2007). 17510360 10.1126/science.1140990

[b14] Bajoni D. *et al.* Polariton Laser Using Single Micropillar GaAs-GaAlAs Semiconductor Cavities. Phys. Rev. Lett. 100, 047401 (2008). 18352332 10.1103/PhysRevLett.100.047401

[b15] Schneider C. *et al.* An electrically pumped polariton laser. Nature 497, 348 (2013). 23676752 10.1038/nature12036

[b16] Lidzey D. G. *et al.* Room Temperature Polariton Emission from Strongly Coupled Organic Semiconductor Microcavities. Phys. Rev. Lett. 82, 3316 (1999)

[b17] Christopoulos S. *et al.* Room-Temperature Polariton Lasing in Semiconductor Microcavities. Phys. Rev. Lett. 98, 126405 (2007). 17501142 10.1103/PhysRevLett.98.126405

[b18] Laussy F. P. Quantum Dynamics of Polariton Condensates in Exciton Polaritons in Microcavities ed. by Timofeev V., Sanvitto D. (Springer-Verlag-Berlin-Heidelberg, 2012).

[b19] Deng H., Weihs G., Santori C., Bloch J. & Yamamoto Y. Condensation of Semiconductor Microcavity Exciton Polaritons. Science 298, 199 (2002). 12364801 10.1126/science.1074464

[b20] Schwendimann P. & Quattropani A. Statistics of the polariton condensate. Phys. Rev. B 77, 085317 (2008).

[b21] Horikiri T. *et al.* Higher order coherence of exciton-polariton condensates. Phys. Rev. B 81, 033307 (2010).

[b22] Lagoudakis P. G., Martin M. D., Baumberg J. J., Malpuech G. & Kavokin A. Coexistence of low threshold lasing and strong coupling in microcavities. Journal of Applied Physics 95, 2487 (2004).

[b23] Tsotsis P. *et al.* Tuning the Energy of a Polariton Condensate via Bias-Controlled Rabi Splitting. Phys. Rev. Applied 2, 014002 (2014).

[b24] Kasprzak J. *et al.* Second-Order Time Correlations within a Polariton Bose-Einstein Condensate in a CdTe Microcavity. Phys. Rev. Lett. 100, 067402 (2008). 18352514 10.1103/PhysRevLett.100.067402

[b25] Wouters M. & Carusotto I. Excitations in a non-equilibrium Bose-Einstein condensate of exciton-polaritons. Phys. Rev. Lett. 99, 140402 (2007). 17930649 10.1103/PhysRevLett.99.140402

[b26] Ciuti C. & Carusotto I. Input-output theory of cavities in the ultra-strong coupling regime: The case of time-independent cavity parameters. Phys. Rev. A 74, 033811 (2006).

[b27] Norris T. B. *et al.* Time-resolved vacuum Rabi oscillations in a semiconductor quantum microcavity. Phys. Rev. B 50, 14663 (1994). 10.1103/physrevb.50.14663 9975704

[b28] Berger J. D. *et al.* The Strong Coupling Threshold. Phys. Rev. B 54, 1975 (1996). 10.1103/physrevb.54.1975 9986047

[b29] Jiang S., Machida S., Takiguchi Y., Yamamoto Y. & Cao H. Direct time-domain observation of transition from strong to weak coupling in a semiconductor microcavity. Appl. Phys. Lett. 73, 3031 (1998).

[b30] Brunetti A. *et al.* Observation of spin beats at the Rabi frequency in microcavities. Phys. Rev. B 74, 241101 (2006).

[b31] Dominici L. *et al.* Ultrafast Control and Rabi Oscillations of Polaritons. Phys. Rev. Lett. 113, 226401 (2014). 25494079 10.1103/PhysRevLett.113.226401

